# Capillary Electrophoresis Separation of Protein Composition of γ-Irradiated Food Pathogens *Listeria monocytogenes and Staphylococcus aureus*

**DOI:** 10.1371/journal.pone.0032488

**Published:** 2012-03-12

**Authors:** Karine Trudeau, Khanh Dang Vu, François Shareck, Monique Lacroix

**Affiliations:** 1 INRS-Institut Armand-Frappier, Research Laboratories in Sciences Applied to Food, Canadian Irradiation Centre, Laval, Québec, Canada; 2 INRS-Institut Armand-Frappier, Laval, Québec, Canada; University of Osnabrueck, Germany

## Abstract

**Background:**

A capillary electrophoresis method using UV detection was developed to analyse protein composition of the lysates of two foodborne pathogens, *Listeria monocytogenes* and *Staphylococcus aureus* which were previously treated at different irradiation doses.

**Methodology and Principal Findings:**

Bacterial samples were γ-irradiated at different doses to produce damage cells, to kill cells and to provoke viable but non culturable cells (VBNC) in order to evaluate the respective expression of stress proteins. In *Listeria monocytogenes*, two proteins (MW of 70.2 and 85.4 kDa) were significantly changed (P≤0.05) at different doses of irradiation. In *Staphyloccocus aureus*, one protein (50 S ribosomal protein) with the MW of 16.3 kDa was significantly decreased at a low dose of irradiation treatment and the other protein (transcriptional regulator CtsR) with the MW of 17.7 kDa was increased significantly (P≤0.05) at all doses of irradiation treatment compared to control.

**Conclusion:**

Expression of two proteins from the acyltransferase family in *Listeria monocytogenes* was statistically changed during irradiation treatment (P≤0.05). In *Staphylococcus aureus*, expression of the 50 S ribosomal protein decreased and the transcriptional regulator CtsR espression increased significantly (P≤0.05) following irradiation treatment. These expressed proteins do not belong to the well-known heat shock proteins family of *Listeria monocytogenes* and *Staphylococcus aureus*. The research further confirmed that capillary electrophoresis is a useful method to separate and analyse proteins expression which may be related to the resistance or sensitivity of food pathogens to γ-irradiation.

## Introduction

According to the Center for Disease Control and Prevention (CDC), food-borne illness is one of the most important health issues in the United States [1]. Foodborne diseases cause approximately 76 million illnesses, 325 000 hospitalizations, and 5 000 deaths in the United States each year [2]. According to the Canada public health experts, there are from 11 to 13 million cases of food-related illness each year. *Listeria monocytogenes* and *Staphylococcus aureus* are two predominant foodborne pathogens. *L. monocytogenes* is the causative agent of listeriosis, a severe disease with high hospitalization and case fatality rates. *L. monocytogenes* can survive and grow in a wide range of environmental conditions such as refrigeration temperatures, low pH and high salt concentrations. This allows the pathogen to overcome food preservation and safety barriers, and pose a potential risk to human health [3]. *S. aureus* is a major agent of gastroenteritis caused by staphyloccal food poisoning following consumption of contaminated food and accumulation of staphylococcal enterotoxins [4].

In order to prevent foodborne illness caused by harmful microorganisms, food irradiation is one of the most effective processes to eliminate foodborne pathogens. Often referred as a cold pasteurization treatment, food irradiation does not cause significant loss of nutrients and sensory qualities in food. Food irradiation utilizes a source of ionizing radiation that passes through food which causes death of harmful bacteria without increasing food temperature [5]. To date, it is still not well established how radiation causes bacterial death, but many theories implied DNA and/or protein damage. The ionizing radiation alters micro-organisms DNA by causing swellings and breakings along the chain. The numerous DNA damage in DNA are merely difficult or impossible to be repaired, thus impairing DNA and bacteria replication leading to death of irradiated microorganisms [6]. In the classical model of radiation toxicity, DNA is the molecule which is the most affected by ionizing radiation. However, recent data show that bacteria survival after irradiation would be more related to the amount of damage occurred to proteins than the quantity of damage done to DNA during irradiation [7].

In a previous study, irradiation treatment of pathogenic bacteria *Staphylococcus aureus* ATCC 29213 and *Listeria monocytogenes* HPB 2812 1/2a at different doses and analysis of expressed Heat shock proteins (Hsps) GroEL by western blot, Caillet et al., demonstrated that radiation treatment significantly increased (P≤0.05) the expression in both bacteria [8]. In *S. aureus*, GroEL concentration was 4.15 µg/mg of total proteins in the control, whereas GroEL level in samples irradiated at 1.2, 2.9 and 3.5 kGy reached 7.98, 10.82 and 8.95 µg/mg of total proteins, representing 1.92-, 2.60- and 2.15-fold increase in the protein expression, respectively. In *L. monocytogenes*, GroEL concentration was 0.83 µg/mg of total proteins in the control and reached 2.56 and 4.79 µg/mg of total proteins in 1.2- and 3.5-kGy samples, respectively. These concentrations were 3.07 and 5.77 times higher compared to control, respectively [8]. Thus it can be demonstrates that irradiation treatment could cause significant changes in protein expression of Hsps in treated pathogenic bacteria.

Protein and peptide analyses are routinely performed using reversed-phase liquid chromatography. The proteins separation is based on hydrophobicity differences between peptides or proteins. Recently, capillary electrophoresis (CE) has increasingly been used for protein and peptide analysis [9]. In capillary zone electrophoresis (CZE), the separation mechanism is mainly based on differences in charge-to-mass ratios. Proteins and peptides are amphoteric, therefore, they are suited to electrophoretic analysis [9]. CE is a modern analytical method that has some advantages such as short analysis time and minimum consumption of both reagents and samples and it is thus suited for the separation of proteins [10]. For understanding more about the composition and function of proteins of organisms, the application of CE in protein analysis increases and therefore, CE has great potential to become one of the key tools in proteome research [10].

It should be emphasized here that beside the changes in the expression levels of Hsps in irradiated cells, it is still not known if there are any changes in the expression levels of other proteins following irradiation treatment. It is expected that the changes in expressed levels (decreases or increases) of other proteins in *S. aureus* and *L. monocytogenes* after irradiation treatment at different doses may led us to speculate about their functions in bacterial survival. Thus, analysis of protein expression profiles in irradiated cells of *S. aureus* and *L. monocytogenes* is necessary. Consequently. the aim of this study was to separate and analyze the protein expression profile in *L. monocytogenes* and *S. aureus* at different doses of γ-irradiation treatment using capillary electrophoresis (CE).

## Results and Discussion

### Assessment of wavelength for detection and quantification of expressed proteins by *L. monocytogenes* and *S. aureus*

**Figure 1:**
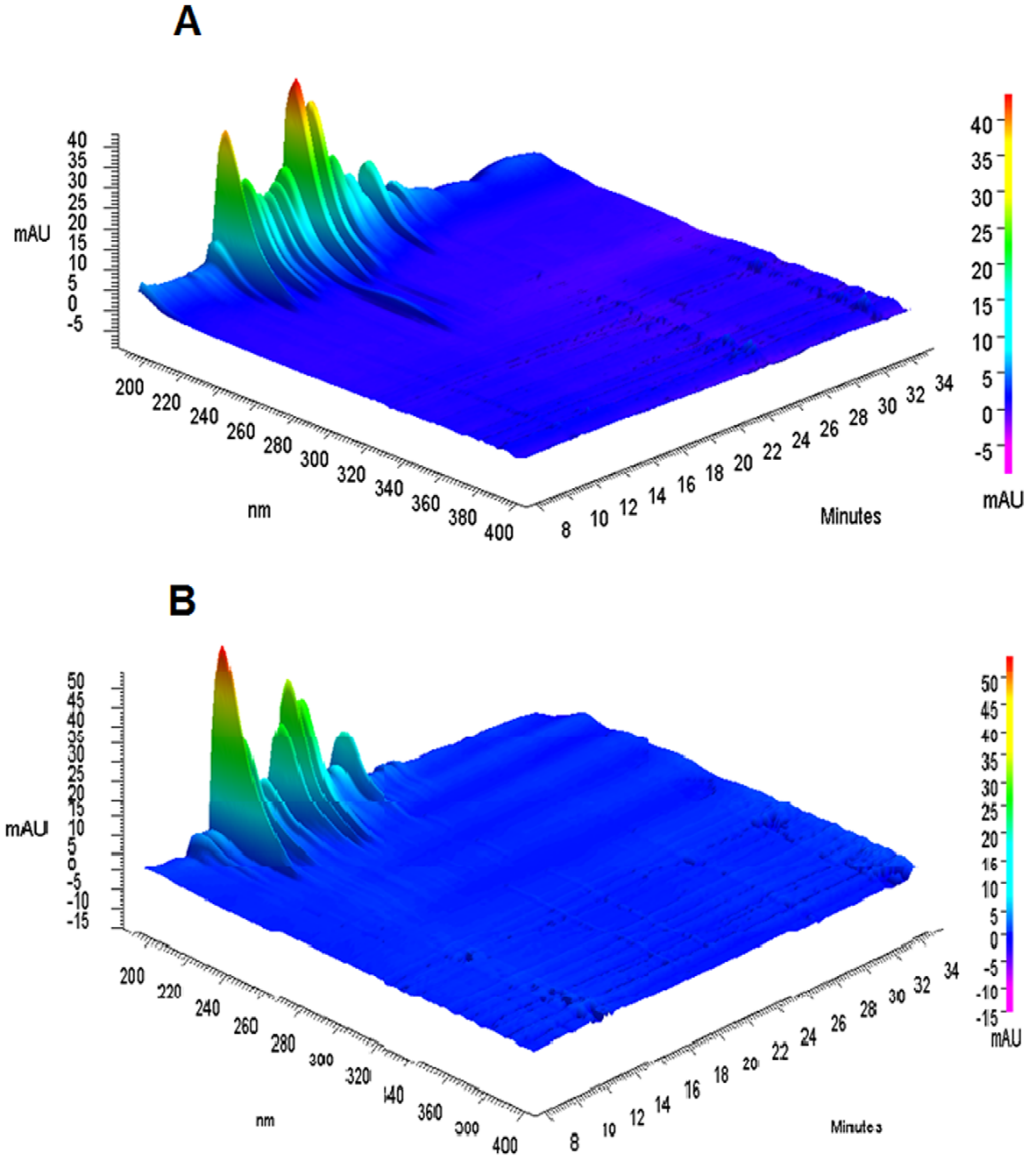
3-D view of absorbance versus time and wavelength (from 190 to 400 nm) for total unirradiated proteins of (A) *L. monocytogenes* and (B) *S. aureus*. Experimental conditions: Bare-fused silica capillary, 30.2 cm (20 cm to the detection window)×50 µm i.d.; temperature, 25°C; applied voltage, 15 kV; electrokinetic injection at 5.0 kV×20 s. Samples were diluted at 1 mg/ml with SDS sample buffer.

Due to the exceptional capabilities of CE, the observed electropherogram depends on the wavelength (WL) chosen for detection of analysed proteins. Although the WL of 280 nm is a characteristic wavelength absorbance for proteins, the minor amount of components in the capillary requires lower WLs of UV detection. Much higher sensitivity can be obtained at the WLs of 200–230 nm, but at any other WLs of UV, the absorbing components might also appear in the electropherogram [11]. It can be observed that there were no other peaks that could be detected at WLs which are longer than 240 nm and there was a very strong absorbance between WLs of 190 and 220 nm (Figure 1. A and B). Our results are also in concordance with the remarks of Huck and Bonn, (2008) and Dolnik (1999) that the WLs between 200 and 220 nm, at which absorption is proportional to the number of peptide bonds, should be chosen for CE [10], [12]. Furthermore, it is known that most of organic solvents have a maximum absorbance at WLs between 180 and 210 nm, thus, it is more reasonable to quantify the expressed proteins in *L. monocytogenes* and *S. aureus* at 220 nm to avoid the interferences caused by alcohol or ethers.

Moreover, to determine the MW of different peaks expressed in an electropherogram, a calibration curve of electrophoretic mobility against the MW was used (data not shown). This calibration curve had a coefficient of determination of 0.998.

### Effects of γ-irradiation on protein expression in *L. monocytogenes*

**Figure 2:**
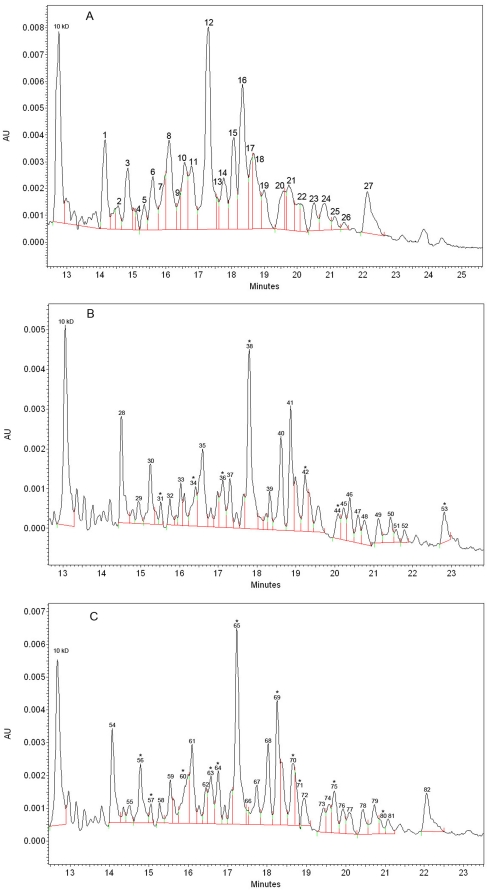
Electrophoretic profiles obtained for *L. monocytogenes* with (A) non-irradiated treatment; (B) irradiated at 1.2 kGy to create cells damaged; and (C) irradiated at 3.5 kGy to kill cells. ^*^mean that corrected area differs statistically between irradiated and non-irradiated proteins by Student's t test with p≤0.05.

The CE electropherograms of expressed proteins in a culture of *L. monocytogenes* treated at different doses of γ-irradiation are presented in Figure 2. The profiles of protein expression in *L. monocytogenes* at different doses of γ-irradiation treatment are presented in Table 1. In this table, the major peaks were calculated and expressed as the equivalent MW and the peak area (%). For non-irradiated bacterial, control), 27 major peaks can be detected (Figure 2.A and Table 1).

**Table 1:** Profiles of protein expression by *Listeria monocytogenes* at different doses of γ-irradiation treatment.

	Peak number and corrected area of protein (%)^a^					
Molecular weigth (kDa)	0 kGy (Control)		1.2 kGy		3.5 kGy	
15.7	1	4.58±0.30	28	4.49±0.20	54	5.16±0.40
17.5	2	0.94±0.20	29	1.46±0.40	55	1.35±0.10
18.8	3	3.46±0.20	30	3.56±0.30	**56** *	4.41±0.30
20.3	4	0.43±0.08	**31** *	0.91±0.06	**57** *	0.96±0.09
21.5	5	1.32±0.30	32	1.12±0.20	58	1.200±0.004
22.7	6	3.37±0.08	33	2.67±1.00	59	2.51±0.20
25.0	7	2.66±0.03	**34** *	3.27±0.20	**60** *	4.88±0.80
25.8	8	6.33±0.40	35	6.52±0.10	61	5.07±0.80
27.7	9	1.13±0.09		ND	62	1.23±0.30
28.5	10	4.13±0.30	**36** *	5.51±0.50	**63** *	2.30±0.70
29.6	11	4.42±0.20	37	4.32±0.80	**64** *	2.89±0.40
33.6	12	14.82±0.60	**38** *	15.92±0.80	**65** *	12.45±0.40
36.2	13	0.57±0.10		ND	66	0.59±0.05
37.4	14	3.47±0.20	39	3.69±0.40	67	3.28±0.03
39.7	15	5.29±0.10	40	5.74±0.40	68	5.29±0.30
42.1	16	10.32±0.40	41	10.63±0.50	**69** *	6.43±0.20
45.3	17	2.75±0.30	**42** *	7.16±0.40	**70** *	4.08±0.50
45.8	18	4.51±0.50		ND	**71** *	1.59±0.40
48.2	19	2.51±0.30	43	2.62±0.40	72	2.09±0.20
53.0	20	2.52±0.30	**44** *	1.90±0.90	73	1.32±0.09
54.1		ND	45	2.58±0.50	74	1.30±0.50
56.0	21	3.72±0.50	46	3.09±0.20	**75** *	2.30±0.40
57.6	22	1.86±0.40	47	1.37±0.50	76	1.25±0.30
60.2		ND	48	1.77±0.30	77	1.20±0.40
64.5	23	1.54±0.10	49	1.53±0.10	78	1.39±0.30
67.9	24	2.33±0.10	50	2.23±0.10	79	1.91±0.50
70.2	25	0.84±0.08	51	0.73±0.02	**80** *	0.47±0.20
71.8	26	0.92±0.20	52	0.53±0.20	81	0.71±0.30
85.4	27	3.91±0.30	**53** *	4.78±0.30	82	3.12±0.50

a The values of corrected area presented are the mean ± standard deviation of samples prepared in duplicate and each injected three times. 0 kGy, control without radiation treatment, 1.2 kGy, damaged cells; 3.5 kGy, lethal. ND is not-determined.

* Corrected area differs statistically from the non-irradiated protein by Student's t test with p≤0.05.

Irradiation at the sensitive dose (1.2 kGy) has caused a significant increase (P≤0.05) in the peak area (%) of different peaks that represented different proteins with the MW of 20.3 kDa (from 0.43% to 0.91%), 25.0 kDa (from 2.66% to 3.27%), 28.5 kDa (from 4.13% to 5.51%), 33.6 kDa (from 14.82% to 15.92%), 45.3 kDa (from 2.75% to 7.16%) and 85.4 kDa (from 3.91% to 4.78%). This treatment only caused a single peak area decrease for a protein with the MW of 53.0 kDa (from 2.52% to 1.90%) (Figure 2.B and Table 1).

More changes in protein expression were found following irradiation treatment at lethal dose (3.5 kGy) of, mainly decrease of the peak area (%), as compared to the sensitive dose. A decrease of expression levels can be found for proteins with the MW of 28.5 kDa, 29.6 kDa, 33.6 kDa, 42.1 kDa, 45.3 kDa, 45.8 kDa, 56.0 kDa and 70.2 kDa. Furthermore, there were also an increase in expression levels of proteins with the MW of 18.8 kDa, 20.3 kDa and 25.0 kDa (Figure 2.C and Table 1).

Although there are 3010 putative proteins encoded in *Listeria monocytogenes 08* – 557 complete genome [13], it can be speculated that only several proteins of *L. monocytogenes* which were treated at different doses of γ-irradiation can be determined based on the molecular weight. Indeed, it is obvious that the whole proteome of *L. monocytogenes* is not expressed at all times. Also, during the extraction, cytosolic proteins were expected to be extracted in larger quantities than membrane proteins. In *L. monocytogenes* [13] many proteins that have approximatively the same MW can be expected but some MWs are represented for unique proteins and are also present in the electrophoretic profiles of our research. It can be observed that at different doses of irradiation treatment, there are three unique proteins (MW of 57.6, 64.5 and 69.7 kDa) whom expression levels were not significantly changed (Table 1). It is presumably expected that these proteins are essential for the survival of *L. monocytogenes* during γ-irradiation treatment.

The protein with a MW of 57.6 kDa is a hypothetical protein LM5578_2803 which was identified in the clinical strain *Listeria monocytogenes* 08-5578 [13]. The protein with a MW of 64.5 kDa is a 2-succinyl-6-hydroxy-2, 4-cyclohexadiene-1- carboxylic acid synthase/2-oxoglutarate decarboxylase (SHCHC) which plays a key role in menaquinone (Vitamin K2) biosynthesis. Menaquinones are constituents of bacterial cytoplasmic membranes. They play important roles in electron transport, oxidative phosphorylation, active transport and endospore formation. Moreover, variations in the inherent structures of menaquinones and their uneven distributions among bacteria are also considered important in bacterial taxonomy [14].

The protein with a MW of 67.9 kDa is a DNA mismatch repair protein (DMR). DMR is a highly conserved biological system that plays a key role in maintaining genomic stability by recognizing and repairing erroneous insertion, deletion and mis-incorporation of bases that can arise during DNA replication and recombination as well as repairing of DNA damages [15].

For some MWs that peaks areas were statistically changed, two proteins can be noticed (Table 1). This is the case of the peak number 53 (Figure 2.B) with a MW of 85.4 kDa. This is a hypothetical protein LM5578_2118 which was identified in the clinical strain *Listeria monocytogenes* 08-5578 [13]. This protein has been found in other *L. monocytogenes* strains and corresponds to formate acetyltransferase. Because there is an increase of this protein at the sensitive dose of irradiation treatment, it can be hypothesised that the treated *L. monocytogenes* cells required more energy production to recover from DNA damages. Finally, the area of peak number 80 (Figure 2.C) with a MW of 70.2 was decreased. This MW corresponds to a hypothetical protein LM5578_1429, which is part of the acyltransferase family proteins in other *L. monocytogenes* strains. At the lethal irradiation dose, the bacterium might require less expression level of this protein which is involved in lipid transport.

The obtained results in this study demonstrate that irradiation treatment at different doses could significantly change the expression levels of proteins or enzymes such as formate acetyltransferase (85.4 kDa, increased at the sensitive dose) and member of the acyltransferase family proteins (70.2 kDa, decreased at the lethal dose) in *L. monocytogenes*. It should be emphasized that the functions of these expressed enzymes are not related to known Hsps such as DnaK, GroES or GroEL. Moreover, in this study, changes in Hsps expression of *L. monocytogenes* were not detected. It is possible that their expression levels were lower than the detection limit of CE. In future researches, *L. monocytogenes* knocked for the proteins explored in this study will be developed in order to better understand their roles in *L. monocytogenes* irradiation treatment responses. Furthermore, other proteomic methods such as Western blot and 2-D electrophoresis will also be used to analyze the proteins composition of *L. monocytogenes* culture sample treated by different doses of irradiation.

### Effects of γ-irradiation on protein expression in *S. aureus*

**Figure 3:**
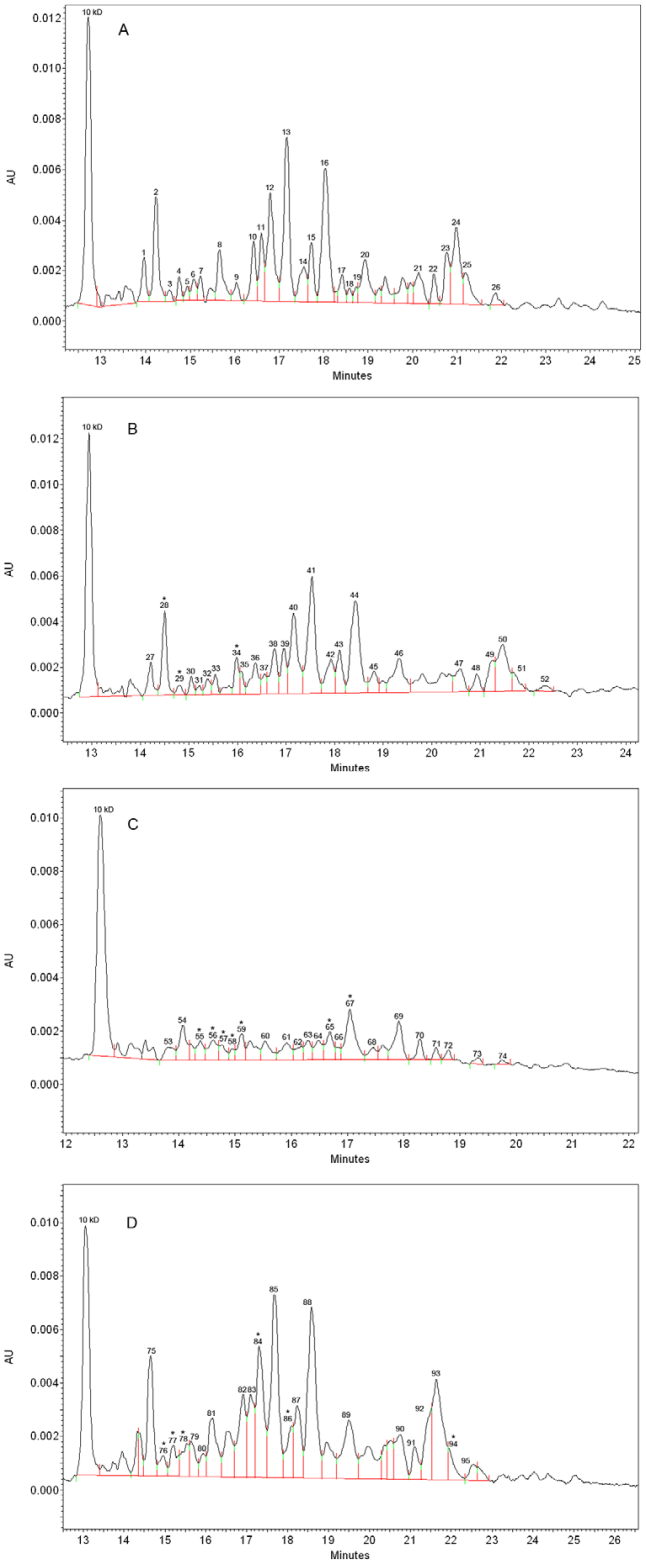
Electrophoretic profiles obtained for *S. aureus* with (A) non-irradiated treatment; (B) irradiated at 1.2 kGy to create damaged cells; (C) irradiated at 2.9 kGy to obtain viable but non cultivable state; and (D) irradiated at 3.5 kGy to kill cells. ^*^mean that corrected area differs statistically between irradiated and non-irradiated proteins by Student's t test with p≤0.05.

The electropherograms of CE of expressed proteins in *S. aureus* treated at different doses of γ-irradiation are presented in Figure 3. The profiles of protein expression by *S. aureus* at different doses of γ-irradiation treatment are presented in Table 2. In this table, the major peaks were calculated and expressed as the equivalent MW and the peak area (%). For the non-irradiated bacteria, control, 26 major peaks were detected (Figure 3.A and Table 2).

**Table 2:** Profiles of protein expression by *Staphylococcus aureus* at different doses of γ-irradiation treatment.

Molecular weight (kDa)	Peak number and corrected area of protein (%)^a^							
	0 kGy (Control)		1.2 kGy		2.9 kGy		3.5 kGy	
15.2	1	2.34±0.05	27	2.40±0.40	53	2.90±0.70		ND
16.3	2	6.20±0.20	**28** *	5.30±0.40	54	6.30±0.10	86	6.80±200
17.7	3	0.61±0.08	**29** *	1.20±0.30	**55** *	3.43±0.01	**87** *	1.20±0.20
18.7	4	1.03±0.05	30	1.40±0.40	**56** *	4.30±0.50	**88** *	1.20±0.50
19.5	5	0.71±0.09	31	0.90±0.10	**57** *	2.30±0.50	**89** *	1.90±0.30
20.3	6	1.30±0.10	32	1.40±0.30	58	1.31±0.06	90	1.30±0.30
21.1	7	1.20±0.10	33	1.30±0.20	**59** *	3.80±0.70	91	1.50±0.70
23.4	8	4.00±0.20	**34** *	2.30±0.10	60	3.90±0.70	92	2.40±2.00
28.2	10	3.70±0.30	38	4.10±0.50	62	3.20±0.20	94	4.57±0.04
29.4	11	3.56±0.04	39	3.90±0.50	63	3.60±0.30	95	3.70±0.50
30.7	12	8.60±0.20	40	8.10±0.30	**65** *	5.15±0.02	**96** *	6.30±0.90
33.4	13	11.6±0.30	41	11.7±0.50	67	11.9±0.40	97	9.60±1.00
36.3	14	3.10±0.03	42	3.00±0.50	**68** *	2.70±0.10	**98** *	2.30±0.20
37.8	15	3.30±0.20	43	3.20±0.50		ND	99	3.40±0.50
40.5	16	11.3±0.30	44	9.70±0.80	**69** *	8.50±0.60	100	9.80±1.00
48.8	20	4.20±0.10	46	1.10±0.20		ND	102	4.60±0.60
61.9	21	2.80±0.20	47	2.50±0.50		ND	106	2.80±0.50
65.9	22	1.63±0.06	48	1.50±0.30		ND	107	1.40±0.30
69.2	23	3.30±0.20	49	2.60±0.50		ND	108	2.60±0.90
72.4	24	6.90±0.30	50	6.60±0.60		ND	109	6.30±1.00
75.1	25	2.76±0.05	51	1.90±0.60		ND	**110** *	1.70±0.50
84.1	26	0.85±0.03	52	0.68±0.03		ND	111	0.85±0.01

a The values of corrected area presented are the mean ± standard deviation of samples prepared in duplicate and each injected three times. 0 kGy, control without radiation treatment; 1.2 kGy, damaged cells; 2.9 kGy, VBNC state just after irradiation; 3.5 kGy, lethal. ND is not-determined.

* Corrected area differs statistically from the non-irradiated protein by Student's t test with p≤0.05.

Irradiation at the sensitive dose (1.2 kGy) has significantly caused an increase (P≤0.05) in the area (%) of the peak representing a protein with a MW of 17.7 kDa (from 0.61% to 1.2%). This treatment also caused decreases in different peak areas (%) that represented proteins with a MW of 16.3 kDa (from 6.2% to 5.3%) and 23.4 kDa (from 4.0% to 2.30%) (Figure 3.B and Table 2).

Irradiation at the VBNC dose (2.9 kGy) affected more the protein expression as compared to irradiation at the sensitive dose (1.2 kGy). It caused increases of peak areas (%) of proteins with an equivalent MWs of 17.7 kDa (from 0.61% to 3.43), 18.7 kDa (from 1.04% to 4.30%), 19.5 kDa (from 0.71% to 2.30%), and 21.1 kDa (from 1.20% to 3.8%). Moreover, this treatment (2.9 kGy) also significantly decreased (P≤0.05) the peak areas (%) of different proteins with the MWs of 30.7 kDa (from 8.6% to 5.15%), 36.3 kDa (from 3.1% to 2.7%) and 40.5 kDa (from 11.3% to 8.5%) (Figure 3.C and Table 2). Explanations for these changes in protein expression following *S. aureus* cells irradiation at 2.9 kGy is not establish. We speculate that some outer membrane proteins increase their expression levels while others proteins impicated in nutrient transport and in ATP synthase decreased their expression levels in VBNC state.

Finally, irradiation at the lethal dose (3.5 kGy), protein expression with a MW of 17.7 kDa, 18.7 kDa and 19.5 kDa were less increased as compared to the VBNC dose (2.9 kGy) and these protein might be very important in the survival of *S. aureus*. Furthermore, there were also decreases in protein expression with a MW of 30.7, 36.3 and 75.1 kDa at this dose of treatment (3.5 kGy) suggesting that these proteins are seriously affected at high dose of γ-irradiation treatment (Figure 3.D and Table 2).

There are 2560 putative proteins identified in *Staphylococcus aureus* subsp. *aureus* USA300_FPR3757 [16], however, we can only speculate the identity of several proteins of *S. aureus* when treated at different doses of irradiation. It can be observed that at different doses of irradiation treatment, there are four unique proteins (MW of 15.2, 69.2, 72.4 and 84.1 kDa) which expression levels were not significantly changed (Table 2). These four proteins are expected to be essential proteins in the survival of *S. aureus* following γ-irradiation treatment. The protein with a MW of 15.2 kDa is a 30 S ribosomal protein S12. This protein is a component of the ribosome, that plays a role in traduction of mRNA into protein [16]. The protein with a MW of 69.2 kDa is a DNA primase which is responsible for synthesizing of RNA primers for initiation of DNA replication [16]. The protein a with the MW of 72.4 kDa is a DNA gyrase subunit B [15]. The protein with a MW of 84.1 kDa is a bifunctional preprotein translocase subunit SecD/SecF, which is part of the preprotein secretory system and stimulates the proton motive force-driven protein translocation [16].

For some MWs that the peak areas were statistically changed, two proteins can be noticed. This is the case of the peak number 2 (Figure 3A) and the peak number 28 (Figure 3B) with the MW of 16.3 kDa which was identified as a 50 S ribosomal protein L9. Considering the decrease expression of this protein, it can be speculated that this protein was more affected by low dose of irradiation treatment. Moreover, as shown in the Table 2, there was an increase in the expression of the protein with a MW of 17.7 kDa and the protein which corresponds to this MW is the transcriptional regulator CtsR [16]. CtsR is a negative heat shock repressor of *clpB*, *clpC* and *clpP* [17]. ClpB and ClpC are expressed proteins required for the growth of *S. aureus* at high temperature and ClpP is an essential protein for the growth of *S. aureus* under heat-shock conditions [17]. In the absence of ClpC and ClpP, CtsR accumulates, so it can be speculated that during the irradiation treatment (at all the irradiation doses) these two proteins (ClpC and ClpP) were degraded and caused the accumulation of the protein CtsR (heat shock repressor). Furthermore, there was a decrease in peak area of the peak number 26 (Figure 3A), when *S. aureus* was treated at the lethal dose (peak 111, Figure 3D) which corresponds to the protein with the MW of 84.1 kDa. This MW correspond to protein quinol oxidase, subunit I [16] which is the terminal oxidase in the respiratory chain of aerobic bacteria and catalyzes the reduction of O_2_.

Thus, the obtained results in this study shows that irradiation treatment at different doses can significantly change the expression levels of proteins or enzymes such as the 50 S ribosomal protein (16.3 kDa, decreased at the sensitive dose of irradiation) and the transcriptional regulator CtsR (17.7 kDa, increased significantly at all doses of irradiation treatment) in *S. aureus*. It should be emphasized that these proteins do not belong to Hsps. Moreover, in this study, changes in the expression of Hsps of *S. aureus* were not detected. It is possible that their expression levels were lower than the detection limit of CE. In future research, *S. aureus* knocked-for the proteins explored in this study will be developed in order to better understand their roles in S. aureus irradiation treatment responses. Furthermore, other proteomic methods such as Western blot and 2-D electrophoresis will also be used to analyze the proteins compositions of *S. aureus* sample treated by different doses of irradiation.

In conclusion, expression protein profiles of *Listeria monocytogenes* sample at different doses of irradiation treatment were studied. There are three unique proteins (MW of 57.6, 64.5 and 69.7 kDa) which expression levels were not significant changed. These proteins could be essentials for the survival of *L. monocytogenes* during γ-irradiation treatment. Moreover, it is also found that expressions of two proteins (MW of 85.4 and 70.2 kDa) of *L. monocytogenes* were statistically changed at different doses of irradiation treatment. The protein with a MW of 85.4 kDa is the enzyme formate acetyltransferase and its expression level was increased at the sensitive dose and the protein with a MW of 70.2 kDa (part of the acyltransferase family proteins) was decreased at the lethal dose.

For *Staphyloccocus aureus*, there were four unique proteins (MW of 15.2, 69.2, 72.4 and 84.1 kDa) which expression levels were not significantly changed at different doses of irradiation treatment. These four proteins could be essentials for the survival of *S. aureus* during γ-irradiation treatment. Moreover, the 50 S ribosomal protein expression corresponding to the MW of 16.3 kDa was significantly decreased at the low dose of irradiation treatment and the transcriptional regulator corresponding to a MW of 17.7 kDa was significantly increased at all irradiated treatments compared to the control.

The research further confirmed that capillary electrophoresis is a useful method to separate and analyse the expressed proteins which may be related to the resistance or the sensitivity of food pathogen to γ-irradiation and therefore, based on the profile of expressed proteins, an advanced approach could be discovered to control food pathogens efficiently.

## Materials and Methods

### Bacterial strains and growth conditions

*Listeria monocytogenes* HPB2812 (Institut National de la Recherche Scientifique - Institut Armand Frappier, Laval, PQ, Canada) and *Staphylococcus aureus* ATCC29213 (American Type Culture Collection, Rockville, MD, USA) were individually sub cultured in tryptic soy broth (TSB; Difco, Becton Dickinson, Sparks, MD, USA) at 37°C for 24 h from the stock cultures maintained at −80°C in TBS containing 20% glycerol. One millilitre of each culture was incubated through two successive incubations of 24 h at 37°C in TSB to obtain approximately 10^9^ CFU ml^−1^. The cultures were centrifuged at 1300× *g* for 15 min., washed with sterilized NaCl 0.85% (w/v) and then resuspended in TSB (500 ml) and incubated at 37°C for 24 h. After the last incubation of 24 h, bacterial cultures were irradiated at suitable doses. In order to obtain a viable but non culturable (VBNC) cells state after irradiation, cell cultures of *S. aureus* were then incubated at 37°C for 5 days to permit the restoration of metabolic activity. Since no VBNC were found for *L. monocytogenes*, this treatment was not applied on this bacterium.

### Irradiation treatment

Bacterial cultures of *S. aureus* were irradiated at different doses of 1.16–1.24 kGy (mean, 1.20 kGy) to create damaged cells (the death ratio was 40% as compared to the control), 2.86–2.94 kGy (mean, 2.90 kGy) to obtain cells under VBNC state (the growth rate was 0 during 5 days of VBNC state) and 3.46–3.54 kGy (mean, 3.50 kGy) to kill cells (the dead ratio was 100% as compared to the control) [17]. The bacterial cultures of *L. monocytogenes* were irradiated at different doses of 1.17–1.23 kGy (mean, 1.2 kGy) to create damaged cells (the dead ratio was 30%) and 3.46–3.54 kGy (mean, 3.5 kGy) to kill cells (the dead ratio was 100% as compared to the control) [18]. An UC-15A irradiator (Nordion International Inc., Kanata, ON, Canada) equipped with a ^60^cobalt source was used to deliver radiation at a dose rate of 18.27 kGy/h. This irradiator was certified by the National Institute of Standards and Technology (Gaithersburg, MD, USA), and the dose rate was established using a correction for decay of source. An Amber Perspex dosimeter 3042D (Atomic Energy Research Establishment, Harwell, Oxfordshire, UK) was used to validate the dose distribution. The radiation treatment was carried out at the Canadian Irradiation Centre (Laval, PQ, Canada) at room temperature (20±1°C).

### Protein extraction

Immediately after the irradiation treatment or after the restoration of metabolic activity in the VBNC state, five hundreds ml of each bacterial culture were quickly chilled in an ice/ethanol bath until the temperature dropped below 10°C. Cells were harvested by centrifugation (Beckman Coulter, rotor: Sorvall GSA) for 15 min at 8670× *g* at 4°C and the resulting pellet was washed with 10 ml of sterilized NaCl 0.85% (w/v). Bacterial pellets were then resuspended in 5 ml of lysis buffer [50 mM Tris-HCl (pH 7.5); 0.1 mM NaCl; 0.5 mM phenylmethylsulfonyl fluoride (PMSF); 1 mg ml^−1^ iodoacetamide] and cell walls were then broken using a cell disrupter (FastPREP, model FP 120, Qbiogene Inc., Carlbad, CA, USA) containing glass beads (0.2 mm) and operated at the speed of 6 m/second for 60 s. Then, the suspension was centrifuged at 2000× *g* for 10 min to remove the glass beads and unbroken cells. Samples were desalted by filtration using Amicon Ultra-4 Centrifugal Units (Millipore, Billerica, MA, USA) as per instructions of the manufacturer.

### Protein quantification

Concentration of proteins extracted from the bacterial cells was quantified using Bicinchoninic acid (BCA) protein assay kit (Pierce, Rockford, IL, USA) and procedure was followed by the manufacturer's specifications. The absorbance was read at 562 nm using a DMS 100 S spectrophotometer (Varian Canada Inc., Mississauga, ON, Canada). Protein concentration of samples was calculated based on standard curve of bovine serum albumin (BSA).

### Capillary electrophoresis

Protein expression was analyzed using a ProteomeLab SDS-Gel MW Analysis Kit and performed on the capillary electrophoresis (CE) system of ProteomeLab™ PA 800 (Beckman Coulter Inc., Fullerton, CA, USA). CE was used due to its many advantages: high speed, automation, low sample consumption. This “dynamic sieving” electrophoresis provides separation of macromolecules according to the molecular weight. A bare-fused silica (30 cm total length, 50 µm i.d.) capillary was used in this study. A commercially available sieving polymer, sodium lauryl sulfate (SDS) was applied for separation of proteins on the basis of their molecular weights. The run buffer of the SDS-Gel Molecular Weight Analysis kit (Beckman Coulter Inc., Fullerton. CA, USA) contains a hydrophilic polymer providing a sieving range from 10, 000 to 225, 000 Da. This polymer was rinsed from the capillary by pressure (5 minutes with 50 psi) between each run using 0.1 M NaOH solution (1 min) and deionized water (2 min). Proteins pellets were resuspended in SDS sample buffer (1% SDS, 100 mM Tris-HCl pH 9.0) to obtain a final concentration of 1 mg ml^−1^. Electrokinetic injection (5.0 kV, 20 s) was used for each sample, the applied voltage was 15 kV (normal polarity) and the temperature of the system was stabilized at 25°C. UV absorption was monitored with a diode array detector at 220 nm and a reference channel at 350 nm, both with a bandwidth of 5 nm. A scan from 190 nm to 400 nm was alsorecorded. An internal standard of 10 kDa was used in each experiment. For estimation of molecular weights of proteins the SDS-Gel Molecular Weight (MW) Analysis Kit (Beckman Coulter Inc., Fullerton. CA, USA) was used. The use of the electrophoretic mobility (μ) instead of the relative migration time is more precise in capillary electrophoresis [9], [10], [12]. Mobility is calculated as the apparent mobility (μ_app_) minus the contribution of electroosmotic flow (μ_eof_). Once the mobility of a reference analyte has been determined, the mobility of related analytes can be calculated using this equation:
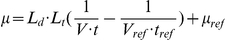
where *V* is the average applied voltage up to the migration time of the peak of interest, *L_d_* is the capillary length to detector, *L_t_* is the total capillary length, *t_ref_* is the migration time of reference peak in the current run, μ*_ref_* is the defined mobility for the reference peak, *V_ref_* is the average applied voltage up to migration time of the reference peak and *t* is the migration time of the peak of interest.

### Protein determination

The strains used in this study, *Listeria monocytogenes* HPB2812 and *Staphylococcus aureus* ATCC29213, have their genomes sequenced and available at (http://www.ncbi.nlm.nih.gov/genomes/MICROBES/microbial_taxtree.html) PubMed, *in silico* studies were done to determine the expressed proteins in *L. monocytogenes* and *S. aureus* based on the MW of detected peak in the electropherograms recorded by CE.

### Statistical analysis

Student's *t* test was utilized and differences between means were considered significant at *P*≤0.05. A Stat-Packets Statistical Analysis software (SPSS Base 18.0, SPSS Inc., Chigaco, Ill.) was used for the analysis. Each sample was prepared in duplicate and injected three times.
